# New methods for improving pancreas preservation

**DOI:** 10.1097/MOT.0000000000001224

**Published:** 2025-05-02

**Authors:** Mohamed A. Elzawahry, Trevor Reichman, Andrew Sutherland

**Affiliations:** a Nuffield Department of Surgical Sciences, University of Oxford, Oxford, UK; Oxford Transplant Centre, Headington, Oxford, UK; b Ajmera Transplant Centre, Toronto General Hospital, University Health Network; Department of Surgery, University of Toronto, Toronto, Ontario, Canada; c Edinburgh Transplant Centre, Royal Infirmary of Edinburgh, Little France Crescent, Edinburgh, UK; Department of Clinical Surgery, University of Edinburgh, Edinburgh, UK

**Keywords:** beta-cell replacement, ischemia-reperfusion injury, islet transplantation, machine perfusion, pancreas transplantation

## Abstract

**Purpose of review:**

Pancreas and islet transplantation face critical organ shortage challenges, with many potential grafts discarded due to concerns about consequences of ischemia-reperfusion injury, particularly from donation after circulatory death (DCD) donors. Static cold storage remains standard practice but has significant limitations. Novel preservation technologies may improve transplant outcomes, donor selection and even expand the donor pool.

**Recent findings:**

Normothermic regional perfusion in DCD donors has increased pancreas utilization with promising one-year graft survival comparable to donation after brain-death (DBD) donors. Hypothermic machine perfusion maintains tissue integrity and shows promising preclinical results. Oxygenated hypothermic machine perfusion successfully restores tissue adenosine triphosphate (ATP) levels without notable tissue injury. Normothermic machine perfusion, despite challenges, offers potential for viability assessment and resuscitation.

**Summary:**

Advanced preservation technologies provide platforms for assessment, reconditioning, and therapeutic interventions for pancreas grafts. Clinical translation requires consensus on perfusion parameters and perfusate composition optimized for pancreatic preservation. Future developments should focus on implementing sensitive and specific assessment methods, including beta-cell specific biomarkers, to confidently select and utilize marginal pancreas grafts for transplantation.

## INTRODUCTION

**FIGURE 1:**
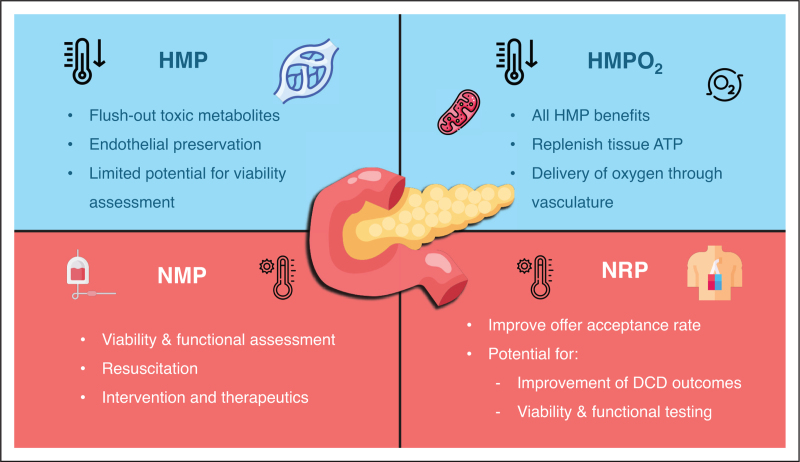
Overview of the potential benefits of using novel methods of preservation applied for pancreas graft preservation.

Beta-cell replacement in the form of pancreas and islet cell transplantation is the treatment of choice for diabetic patients with severe and life-threatening complications such as renal failure and hypoglycaemic unawareness. Both offer excellent outcomes for these patients with over 90% graft survival at one year [1,2]. However, transplant volumes are limited by the availability of quality organs. The fear of ischaemia-reperfusion injury (IRI) that occurs after transplantation is one of the main reasons for declining marginal pancreases, especially in the case of donation after circulatory death (DCD) [3,4]. Less than 64% of donation after brain-death (DBD) and 55% of DCD retrieved pancreases are used for transplant in the UK [5]. By improving preservation and utilizing new technologies to assess and recondition pancreases, the risk of pancreas transplantation can potentially be minimized while improving the number and outcomes of transplants. This review focuses on advancements in new technologies for pancreas preservation, illustrated in Fig. 1. 

**Box 1:**
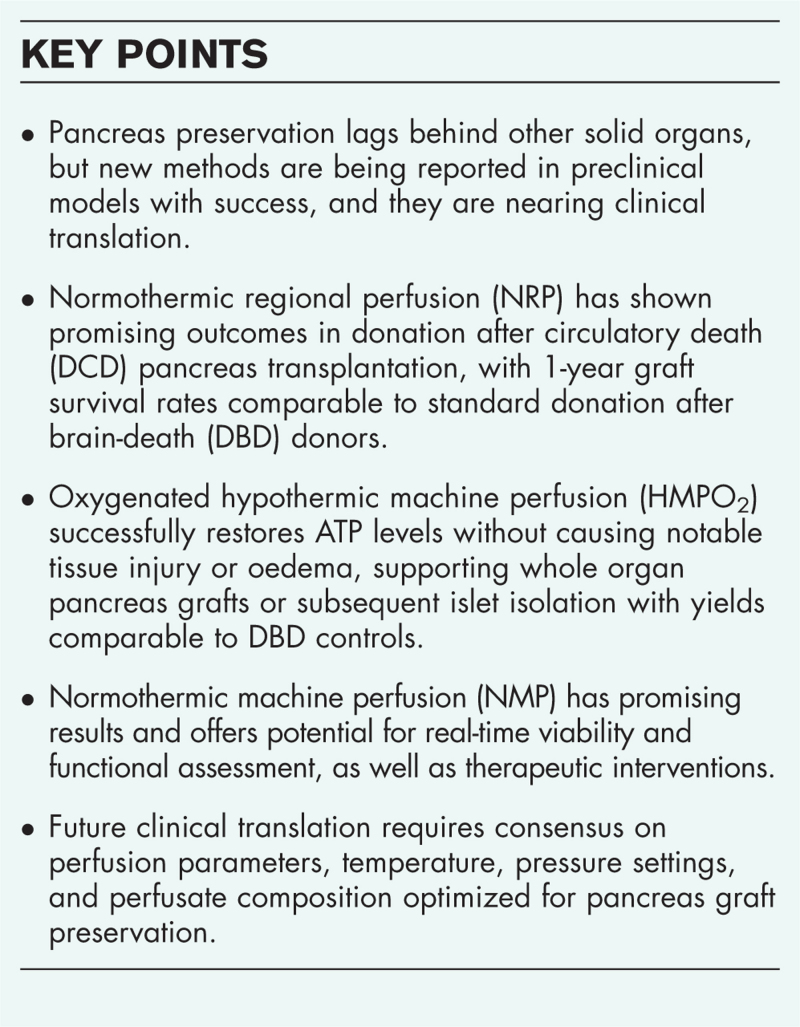
no caption available

## THE CURRENT STANDARD OF PANCREAS PRESERVATION

The current standard remains static cold storage (SCS) with cold-preservation solution [typically University of Wisconsin (UW) solution [6]] on ice. The recovery and transplant process generates a sequence of insults: warm ischaemia, cold ischaemia, rewarming and reperfusion with recipient blood, which result in ischaemia-reperfusion injury (IRI). During SCS, adenosine triphosphate (ATP) is depleted, and anaerobic metabolites, (e.g., succinate and lactate) accumulate leading to ionic pump failure, acidosis, reactive oxygen species (ROS) production, and antioxidant depletion [7]. Upon transplantation, oxygen delivery resumes, resulting in a burst of ROS, an inflammatory cascade and cell injury, leading to endothelial dysfunction and microcirculatory failure [8–13].

In whole-organ pancreas transplants, IRI manifests as graft pancreatitis [14–16] potentially leading to graft loss and life-threatening morbidity. In islet transplantation, IRI results in decreased islet yield, functional quality and viability after implantation [17,18].

SCS remains the gold standard because of its ease, affordability, and sufficient effectiveness. However, it prevents flushing of toxic metabolites, provides insufficient oxygen and nutrient provision for residual metabolic activity.

## NORMOTHERMIC REGIONAL PERFUSION

Pancreas and islet transplantation from DCD donors have increased since similar graft and patient outcomes were demonstrated as comparable to DBD donors in the United States [3,19] and the UK [15]. However, more selective acceptance of DCD organs and higher discard rates from DCD donors have been identified as challenges [15].

Normothermic regional perfusion, an in-situ preservation technology, entails recirculating, extracorporeally oxygenated, warm blood in the abdominal or thoraco-abdominal regions of DCD donors after cessation of circulation. This is established as standard clinical practice in some European countries and its use is expanding in North America. Applying this preservation modality has improved outcomes in the recovery of DCD livers [20–23] and kidney for transplantation [24] but its benefits on pancreas and islet transplantation are still uncertain. There are only a few reports documenting pancreas and islet transplantation in the UK [25], Spain [26,27], and the United States [28].

Even though there is a widely held belief that NRP is beneficial for pancreas preservation, the data is limited. Outcomes from the UK show a 1.6-fold increase in the transplantation of DCD pancreases with the use of NRP, and there was a trend towards improvement in 1-year graft survival [25,29]. In addition, the UK reported significantly lower serum lipase, but not amylase, levels in the 13 recipients of normothermic regional perfusion (NRP)-DCD grafts when compared to standard DCD, which may indicate reduced IRI [30]. It remains challenging to draw firm conclusions with such small numbers, but the indication is that it is at least comparable to DBD outcomes, potentially avoiding the higher morbidity burden associated with DCD pancreases [23,29,31]. This view has been reinforced by data presented at the recent European Pancreas and Islet Transplant Association (EPITA) symposium [32] which reports the outcomes of 36 simultaneous kidney pancreas transplants following NRP retrieval compared to contemporaneous standard DCD controls. 1-year pancreas survival was higher in the NRP group (93.6%) compared to standard DCDs (89.2%) but this did not reach statistical significance (*P* = 0.206).

Also, there are limited but promising reports of the use of NRP prior to islet transplantation. Two islet isolations after NRP were reported in the UK, one having insufficient islet yield as it was fibrotic and the second leading to transplantation [23]. Recently, the Leiden group in the Netherlands reported 10 research islet isolations and 1 clinical case with transplantation, demonstrating successful isolation with significantly higher islet yields than standard DCD donors. [33^▪^]

Currently, the limitation of NRP is a lack of functional and/or viability assessment during pancreas retrieval. However, liver assessment during NRP has been reported using surrogate markers of graft function [34].

## TWO-LAYER METHOD & PERSUFFLATION

The two-layer method (TLM) attempts to diffuse oxygen into the pancreas while suspended between two discrete layers: a conventional organ preservation solution, and a perfluorocarbon (PFC) [35], which can reversibly bind to oxygen with much greater capacity than a simple solution [36]. Despite promising results in a canine model [37], clinical experience is limited to one report of TLM being equivalent to SCS in a cohort study that was not sufficiently powered and included a heterogeneous group of whole organ pancreas transplant recipients [38]. The effectiveness of TLM is limited to smaller, thinner pancreases and was not demonstrated in larger animal or human models. To circumvent TLM's limitations, oxygen delivery through the vasculature can be an effective alternative [39].

Persufflation (PSF) perfuses the organ vasculature with humidified oxygen. It has been tested in heart [40], liver [41], kidney [42], small intestine [43], and pancreas transplantation [44,45]. Porcine and DBD human pancreas models showed its effectiveness in tissue oxygen delivery and restoration of ATP levels using nuclear magnetic resonance spectroscopy [45]. Its application for pancreas preservation particularly for islet isolation has potential but direct evidence is yet to be reported.

## EX-VIVO MACHINE PERFUSION

Ex-vivo machine perfusion (MP) is the continuous or pulsatile pumping of a blood-based or simple solution, through the vasculature of a graft either at a low temperature (hypothermic machine perfusion, HMP < 12°C), below body temperature (sub-normothermic 25–34°C) or at body temperature (normothermic machine perfusion, NMP, aiming at 37°C).

The application of ex-vivo MP in the preservation of other solid organs has gone much further than pancreas in the last two decades and includes kidney [46,47], liver [48,49], heart [50] and lungs [51].

The reported benefits of MP are due to:

(1) Flushing-out of toxic metabolic byproducts.
(2) Maintaining the integrity of the microvasculature and as a result reducing vascular resistance.
(3) Delivering oxygen and nutrients
(4) Replenishing ATP
(5) using MP as a platform for therapeutic interventions and viability, as well as functional testing.

## HYPOTHERMIC MACHINE PERFUSION

Nonoxygenated HMP is the standard of care in kidney preservation in many European countries and North America. A multicentre randomised controlled trial (RCT) of continuous HMP (started at retrieval) showed benefit over SCS [52] and reduced delayed graft function especially in marginal grafts [53] and DCD kidneys [46]. Contrasting evidence from a UK RCT showed no advantage of employing HMP after a period of SCS (end-ischaemic) [54].

HMP of pancreas grafts has only been assessed in preclinical models, summarised in Table 1. In a porcine DCD model, end-ischaemic HMP produced an increase in weight and stable histological features [55]. 5 h of end-ischaemic HMP of three porcine and three human pancreases after 26 h of SCS was compared to 26 h of SCS. There was significant weight gain in all pancreases. Two human and two porcine end-ischaemic HMP pancreases, and none of the SCS pancreases, demonstrated insulin response to glucose stimulation. Histologically no changes were noted after HMP [56]. A series of porcine pancreas allotransplants in diabetic pigs after HMP preservation for 6 h reported no evidence of pancreas vascular thrombosis [57].

**Table 1:** Summary of the reported evidence for the use of HMP in preservation of pancreas grafts

Author	Year	Species	Donor type	Mode of perfusion	Pressure (mmHg)	Device	Perfusate	Control	Sample size (*n*)	WIT; CIT (mins)	Perfusion time (hours)	Assessment	Key conclusions
Karcz *et al.*[55]	2010	Porcine	DCD	End-ischaemic HMP	20	RM3	UW solution	None	15	25; 150	5.25	Histological	Increase in weight and stable histological features.
Taylor *et al.*[60]	2010	Porcine	DBD	Continuous	10	Lifeport kidney transporter	KPS1 solution	SCS (24h and <2h CIT)	14	0 (*n* = 7), 30 (*n* = 7)	24	Histology, Islet isolation and functional assessment	Islet isolation was feasible producing greater yield & purity of islets after HMP.
Hamaoui *et al.*[56]	2017	Porcine and human	DCD	End-ischaemic	30	RM3	UW	SCS (porcine)	3 porcine + 3 human	30–55; 26	5	NR & GSIS	Graft weight gain but no histological changes after HMP. 4 out of 6 HMP pancreases showed insulin response, but none of the SCS.
Branchereau *et al.*[59]	2018	Human	Not reported	End-ischaemic	25	Waves	Perf-Gen	SCS	7	–	24	Histology, perfusion parameters & macroscopic oedema score	Minimal oedema. No further histological deterioration after HMP, but ischemic necrosis after 24h of SCS.
Prudhomme *et al.*[58]	2020	Baboon	DBD	Continuous	15, 20 & 25	Waves	IGL-1	SCS (IGL-1)	5	–	24	Biochemistry and histology	No histological evidence of apoptosis at 24 h, assessed as <1% cleaved caspase 3 activity.
Prudhomme *et al.*[57]	2021	Porcine	DBD	Continuous	–	Waves	IGL-1	SCS (IGL-1)	8	–	6	Allotransplantation	No evidence of pancreas vascular thrombosis

DBD, donation after brain-death; DCD, donation after circulatory death; NMP, normothermic machine perfusion; NR, normothermic reperfusion; NRP, normothermic regional perfusion; SCS, static cold storage.

HMP of nonhuman primate pancreases was comparable to SCS with no histological evidence of apoptosis at 24 h [58]. HMP of seven human pancreases declined for transplantation and four in SCS showed minimal or no oedema macroscopically. On histology, two of the HMP group showed evidence of fat necrosis from the start but no further deterioration, compared to findings of ischaemic necrosis in the SCS biopsies at 24 h [59].

Islet isolation from porcine pancreases after 24-h preservation with HMP was reported as feasible, despite the moderate oedema [60].

## OXYGENATED HYPOTHERMIC MACHINE PERFUSION

HMPO_2_ combines hypothermia's safety, dynamic machine perfusion and oxygen delivery to support mitochondrial function to mitigate the effects of IRI. HMPO_2_ reduced postreperfusion syndrome, early allograft dysfunction, and the incidence of symptomatic nonanastomotic biliary strictures in an RCT following liver transplantation [48]. When HMPO_2_ was compared to HMP in kidney preservation, it was associated with fewer biopsy-proven rejection episodes and a lower rate of graft failure [61].

The earliest reports of HMPO_2_ in pancreas preservation were in canine transplant models, with mixed results and inconsistent methods. However, researchers attempted to test different perfusion pressures and perfusate compositions, delivering oxygen with varying methods [62–64].

More recently, multiple NMP reperfusion models were used to assess porcine pancreases after HMPO_2_ as compared to SCS. These studies highlighted improved perfusion parameters, less parenchymal oedema, comparable markers of damage and better functional assessment in the form of glucose stimulated insulin secretion, with continuous HMPO_2_ using UW [65,66^▪^]. A similar porcine model showed that HMPO_2_ can provide tissue oxygenation, increasing tissue ATP levels without the need for perfusate oxygen carriers [67^▪▪^].

Recently, a human split-pancreas model compared SCS, HMP and HMPO_2_ from 15 DBD and 9 DCD donors. Islet isolation was performed with comparable results across all groups, in both DBD and DCD pancreases [68].

HMPO_2_ of 10 declined human pancreases demonstrated homogeneous perfusion and an increase in tissue ATP concentration as compared to SCS, supporting the hypothesis that oxygen delivery restores cellular energy stores under hypothermic conditions. No evidence of cellular injury, oedema, or ROS was noted after 6 h of perfusion. Two pancreases underwent islet isolation, confirming islet viability and function [69].

Five declined human DCD pancreases were preserved for 6 h by HMPO_2_ after 7.4 h of SCS. No evidence of oedema or apoptosis on biopsy was noted after HMPO_2_, and subsequent islet isolation produced similar yield, purity, and function of islets to DBD controls in SCS [70].

Mounting evidence for the feasibility and benefit of HMPO_2_ from experimental studies, summarised in Table 2, warrants a safety and feasibility clinical trial. A first-in-human NIHR funded trial is yet to recruit its first patient in the UK, at the time of authoring this review.

**Table 2:** Summary of the reported evidence for the use of HMPO_2_ in preservation of pancreas grafts

Author	Year	Species	Donor type	Mode of perfusion	Pressure (mmHg)	Device	Perfusate	Control	Sample size (*n*)	WIT; CIT (mins)	Perfusion time (hours)	Assessment	Key conclusions
Leemkuil *et al.*[69]	2018	Human	5 DBD and 5 DCD	End-ischaemic	25	Dual XVIVO kidney transporters	UW-MPS	SCS	10	DCD 18; 4 DBD -; 3	6	Histology, Islet isolation	Increase in tissue ATP concentration contrary to SCS. No evidence of cellular injury, oedema, or ROS. 2 underwent islet isolation, confirming feasibility.
Doppenberg *et al.*[70]	2021	Human	DCD	End-ischaemic	25	Dual XVIVO kidney transporters	UW-MPS	DBD SCS	5	18; 7.4	6	Histology, islet isolation, in-vitro & in-vivo functional assessment	No evidence of oedema or apoptosis. Islet isolation produced similar yield, purity, and function of islets to DBD controls in SCS.
Ogbemudia *et al.*[65]	2021	Porcine	DCD	End-ischaemic	15	Waves	UW-MPS or IGL-2	SCS (*n* = 4)	9	25; 3	6	NR and GSIS	Improved perfusion parameters, less parenchymal oedema after HMPO_2_, and better glucose stimulated insulin secretion using UW- MPS.
Elzawahry *et al.*[66^▪^]	2023	Porcine	DCD	Continuous vs. end-ischaemic	25	Xvivo kidney transporter	UW MPS	SCS (*n* = 6)	12	18.7; 8.7 (SCS) & 6.7 (end-ischaemic)	8.7 (continuous, n = 6) & 2 (end-ischaemic, n = 6)	NR and GSIS	Improved perfusion parameters, and significantly higher glucose stimulated insulin secretion with continuous HMPO_2_
Buemi *et al.*[68]	2024	Human (split)	15 DBD and 9 DCD	End-ischaemic	25	Lifeport kidney transporter	UW-MPS	SCS	24	23.5; 18	DCD (HMP 13.4, HMPO2 11.7) & DBD (HMP 16.4, HMPO2 17.5)	Histology, Islet isolation and in-vitro functional assessment.	Islet isolation was feasible with comparable purity and function to SCS.
Mesnard *et al.*[67^▪▪^]	2025	Porcine	DCD	Continuous	15	Waves	IGL-1 ± M101	SCS (*n* = 4), HMP (*n* = 4)	24	30; -	24	NR and biochemistry.	HMPO_2_ increases tissue ATP levels without oxygen carriers.

DBD, donation after brain-death; DCD, donation after circulatory death; NRP, normothermic regional perfusion; NMP, normothermic machine perfusion; SCS, static cold storage.

## NORMOTHERMIC MACHINE PERFUSION

Normothermic machine perfusion (NMP) has been the focus of organ preservation research since the inception of transplantation. NMP aims to preserve organs in their physiologic, active state by providing oxygen and nutrients to maintain cellular function. This offers an advantage over HMP aiming to allow viability assessment, functional testing, reconditioning, and therapeutic interventions of the graft. NMP has been successfully implemented into clinical practice for liver, heart, lung, and kidney. In liver, NMP has increased graft utilization and lowered early graft injury [71]. Lung NMP allowed for doubling the number of lungs transplanted annually [72]. In kidney, Nicholson and Hosgood demonstrated reduced delayed graft function in extended criteria donor (ECD) kidneys undergoing NMP [47]. Recently, a clinical study showed it is safe to extend kidney NMP preservation up to 24 h [73]. However, NMP as a tool for pancreas preservation has been limited to experimental, preclinical models with the earliest application as a preclinical assessment tool to mimic early post transplantation IRI and acute pancreatitis [74].

The preclinical models of pancreas NMP, summarised in Table 3, have not seen significant advancement since the early reports [75,76] over 4 decades ago. Success has been limited due to graft oedema, haemorrhage, severe inflammation, intra-vascular thrombosis and necrosis and only limited functional response to glucose/arginine stimulation. This is in spite of varying perfusion pressure, perfusate composition, use of dialysis or simultaneous kidney perfusion, porcine and human models, and use of DBD/DCD [77–79]. The longest successful pancreas NMP reported to date was a single human DBD pancreas preserved for 12 h on NMP after 4 h of SCS – this showed a reassuring histological picture. C-peptide was detected during NMP, but stimulation testing was not performed [80].

**Table 3:** Summary of the reported evidence for the use of NMP in preservation of pancreas grafts

Publication	Year	Species	Donor type	Mode of perfusion	Pressure (mmHg)	Device	Perfusate	Control	Sample size (*n*)	WIT;CIT (mins)	Perfusion time (hours)	Assessment	Key conclusions
Barlow *et al.*[77]	2015	Human	3 DBD and 1 DCD	End-ischaemic	50-55	Cardio-pulmonary bypass technology	Packed RBCs + Gelofusine + mannitol	None	4	-; 15.5	1 (*n* = 1) & 2 (*n* = 3)	Perfusion parameters & histology	NMP pancreas is feasible, but requires significant refinement for use in preservation
Kuan *et al.*[78]	2017	Porcine	DBD	Continuous	70-80	Roller pump + paediatric ECMO oxygenator	Whole blood	None	4	8.25; 34	1–1.5	Perfusion parameters & histology	It was feasible but for a short period as a method of assessment and not preservation.
Kumar *et al.*[79]	2018	Porcine	DCD	End-ischaemic	20 (*n* = 4) & 50 (*n* = 9)	Cardio-pulmonary bypass technology	Whole blood	None	20 mmHg (4) vs. 50mmHg (9)	4.5; 131.5	3	Perfusion parameters & histology	No difference between standard and low perfusion pressure.
Nassar *et al.*[80]	2018	Human	DBD	End-ischaemic	60	-	Packed RBCs and plasma.	None	3	-; 4	6 (*n* = 2) and 12 (*n* = 1)	Histology	Report of a 12-h successful pancreas NMP.
Mazilescu *et al.*[81]	2022	Porcine	DCD	Continuous	25	S3 heart-lung machine + neonatal cardio-pulmonary bypass	packed RBCs + STEEN Solution + aprotinin	None	10	10; -	3 (*n* = 3) & 6 (*n* = 7)	Histology, biochemistry and Allotransplantation.	Transplantation was feasible, with immediate and excellent graft function (glucose tolerance).
Parmentier *et al.*[83]	2023	Human	5 DBD and 1 DCD	End-ischaemic	15-25	S3 heart-lung machine + neonatal cardio-pulmonary bypass	packed RBCs + STEEN Solution + aprotinin	None	6	17 (DCD); 372.5	4	Perfusate, histology	NMP was feasible, maintaining both macroscopic and microscopic appearances.
Parmentier *et al.*[84]	2023	Human	DBD	End-ischaemic	15-25	S3 heart-lung machine + neonatal cardio-pulmonary bypass	packed RBCs + STEEN Solution + aprotinin	None	2	-;7.75	4	Histology and islet isolation	Islet isolation after NMP is feasible using Edmonton protocol.
Ray *et al.*[82^▪^]	2024	Porcine	DCD	End-ischaemic	15-25	S3 heart-lung machine + neonatal cardio-pulmonary bypass	packed RBCs + STEEN Solution + aprotinin	SCS	6	-; 21-24	3	Histology, allotransplantation, functional assessment	Demonstrated benefit of resuscitating grafts damaged by prolonged cold ischemia using NMP.

DBD, donation after brain-death; DCD, donation after circulatory death; NRP, normothermic regional perfusion; NMP, normothermic machine perfusion; SCS, static cold storage.

More recently, further success was reported on a healthy porcine model with a brief period of SCS. Seven pancreases were subjected to NMP for 6 h at 25 mmHg with an in-circuit dialysis and serine protease inhibitor as adjuncts. These organs demonstrated stable perfusion parameters, biochemical homeostasis and only mild graft injury. In the same study, three pancreases were preserved for 3 h and successfully transplanted into recipient pigs following a native pancreatectomy. These pigs were then observed for 48–72 h and maintained physiologic glucose levels and responded appropriately to a glucose challenge [81]. Furthermore, NMP was tested for its ability to recondition pancreas grafts after prolonged cold ischaemia (i.e. 21 h of SCS). Using a porcine allotransplant model, a significant survival benefit was demonstrated in porcine grafts treated with NMP as compared to SCS alone [82^▪^].

Using a similar NMP protocol in humans, the same group perfused human pancreases (5 DBD and 1 DCD) declined for transplantation for 4 h after a mean of 6.2 h of SCS. They were able to validate their findings of stable perfusion and biochemical parameters, minimal tissue injury, and recorded insulin and c-peptide secretion [83]. Furthermore, the feasibility of isolating functional islets from after 4 h of NMP was demonstrated in two discarded human pancreases [84].

## CONCLUSIONS AND FUTURE DIRECTIONS

The major obstacle facing pancreas and islet transplantation is the stringent selection criteria of suitable pancreas donors making an already insufficient pool of donors even smaller. Currently, the risk of poor outcomes from marginal donors is driven by the lack of better preservation methods and accurate assessment tools to select appropriate extended criteria grafts.

Novel methods of preservation provide a platform for assessment, reconditioning and even therapeutics for pancreas grafts. All the new methods of pancreas preservation discussed above, need stronger evidence to support their incorporation into routine clinical use. To provide a path forward, more consensus on perfusion modality, temperature, pressure and flow rate settings is needed. In addition, perfusate composition, especially adjuncts, should be further defined to allow for safe reproducible perfusion, particularly in NMP [85].

The hope is that the future of pancreas preservation will lead to better assessment of grafts and potentially therapeutic intervention. Unfortunately, to date, the tools used in the studies described above only reflect the degree of graft injury and rudimentary test of endocrine function. New, more effective biomarkers are needed for the pancreas that can more effectively predict post IRI and early/late graft function. With better preservation and assessment tools, the pool of marginal grafts can be more safely and effectively utilized to provide beta cell replacement to the growing population of patients with diabetes.

## Acknowledgements

*Mohamed Elzawahry is salaried through project funding from the NIHR (NIHR204643).*

*The figure has been designed using resources from Flaticon.com.*

### Financial support and sponsorship

*None.*

### Conflicts of interest

*There are no conflicts of interest.*

## REFERENCES AND RECOMMENDED READING

Papers of particular interest, published within the annual period of review, have been highlighted as:

▪ of special interest
▪▪ of outstanding interest
